# Care Pattern for Fontan-Associated Liver Disease by Academic Pediatric Hepatologists in Canada

**DOI:** 10.1097/PG9.0000000000000207

**Published:** 2022-06-21

**Authors:** Mohit Kehar, Carolina Jimenez-Rivera

**Affiliations:** From the Division of Pediatric Gastroenterology, Hepatology, and Nutrition, Children’s Hospital of Eastern Ontario, Department of Pediatrics, Faculty of Medicine, University of Ottawa, Ottawa, Ontario, Canada.

**Keywords:** pediatric, cardiac, hepatic disease, Canada, congenital heart disease

## Abstract

Fontan-Associated Liver Disease (FALD) is a common extracardiac complication seen in patients following the Fontan procedure. There are no consensus guidelines on screening and management of children with FALD.

**Objective:**

The current study aims to determine academic pediatric hepatologists’ practices and identify variability in management provided to children with FALD in Canada.

**Methods:**

Using the infrastructure of the Canadian Pediatric Hepatology Research Group, a nationwide survey was distributed electronically to all pediatric hepatologists practicing in university-affiliated hospitals.

**Results:**

Twelve pediatric hepatologists from 12 of 13 academic centers (92%) responded to the survey. The institutions of only 2 (17%) physicians offer post-Fontan care with a multidisciplinary team, both from different provinces. The screening for other comorbidities, use of noninvasive modality, and timing of liver biopsy for estimation of liver fibrosis and screening for esophageal varices differ from program to program. The frequency of outpatient clinic follow-up varies significantly. Education and counseling concerning liver health are generally used as treatment; only 58% of academic centers have a formal adult care transition plan.

**Conclusions:**

Significant discrepancies exist in the care provided to children with FALD by hepatologists practicing in academic centers across Canada. Future study is needed to develop a standardized protocol for managing and following children and youth with FALD.

**What Is Known**

- Fontan-Associated Liver Disease is a common extracardiac complication seen in patients after Fontan Procedure.
- Liver fibrosis is universally present in children by adolescent age.

**What Is New**

- Significant variability is seen in clinical practice in the management of children with Fontan-Associated Liver Disease in Canada.
- Frequency of follow-up, evaluation, screening for complications varies amongst academic hepatologists in Canada.
- Multidisciplinary team care is uncommon in the management of children with Fontan-Associated Liver Disease in Canada.

## INTRODUCTION

Fontan procedure introduced in 1971 as palliation for patients with tricuspid atresia is now the preferred strategy for various single ventricle congenital heart diseases.^1,2^ The procedure separates the pulmonary and systemic circulation and allows the systemic venous return to the pulmonary arteries directly hence bypassing the right ventricle. In the United States, over 1000 children undergo the Fontan procedure every year.^3^ With the refinement in surgical procedure, most patients post-Fontan procedure are surviving into adulthood.^4,5^ Long-term survivors are at high risk for late cardiac decompensation as well as extracardiac complications.

Fontan-Associated Liver Disease (FALD) is one of the most common extracardiac complications associated with raised central venous pressure (CVP), causing chronic hepatic congestion together with reduced hepatic blood flow caused by low cardiac output.^6^ Despite increasing awareness of FALD in adults^7^ and in children, the literature on FALD in children consists of single-center studies,^8–10^ and previous efforts to use administrative databases to analyze the epidemiology have proven ineffective.^11^ The number of Fontan procedures performed in Canada is unknown, however, from 2012 to 2018, 532 infants (532/804 292 singletons) from Ontario were born with single ventricle congenital heart disease in a recent population-based study which is often the indication for a Fontan procedure.^12^ Studies have shown that a fraction of patients has hepatic abnormalities seen even before the Fontan procedure^13^; moreover recent single-center studies suggest that virtually 100% of patients post-Fontan have some degree of hepatic fibrosis, with bridging fibrosis reported in nearly 40% by adolescence.^8,9^ Bridging fibrosis had been associated with worse survival in adolescents with FALD.^14^ Despite scarce data on the progression and natural history of liver disease in patients with Fontan physiology, advanced liver disease and hepatocellular carcinoma (HCC) with poor prognoses have been reported in children as well as in adults.^15–19^ In adult patients, decompensation events including gastroesophageal varices,^20,21^ encephalopathy,^22^ and ascites^7^ are reported, with poor outcomes.

FALD screening and management guidelines are lacking in the pediatric population despite growing evidence of advanced liver fibrosis, HCC, and poor quality of life.^23^ No data are available on current practice patterns in Canada in patients with suspected FALD in children or adults. We aimed to examine the clinical care practices of pediatric hepatologists practicing in academic hospitals in Canada for patients with FALD.

## METHODS

### Study Design

We conducted an online anonymous Redcap survey that was sent to all academic pediatric hepatologists using the infrastructure of the Canadian Pediatric Hepatology Research Group from September 2021 to December 2021. Canadian Pediatric Hepatology Research Group is a research collaborative group of all academic pediatric hepatologists practicing across 13 academic pediatric centers in Canada. Participants were contacted via e-mail to voluntarily participate in the survey. Consent to participate was implied and 2 reminders were sent 2 weeks apart. No compensation was provided for survey completion. In cases with more than 1 hepatologist working at any center, only 1 completed the survey to avoid duplication of center’s data. We used the Checklist for Reporting Results of Internet E-Surveys guidelines for reporting the results (See Supplemental Digital Content Table 1, http://links.lww.com/PG9/A84).^14^ The study was approved by the Research Ethics Board at Children’s Hospital of Eastern Ontario.

### Study Objectives

To determine the clinical care practices and the variability in the care of children and adolescents with FALD among pediatric hepatologists practicing in Canadian academic pediatric hospitals.

### Survey Development and Data Collection

A 48-item questionnaire was developed to assess the clinical care practice patterns of pediatric FALD (See Supplemental Digital Content Table 2, http://links.lww.com/PG9/A84). The survey was divided into 6 parts: respondent information, referral status and care pattern, monitoring, assessment, treatment patterns, and transition of care. Survey items include open text, scales, yes/no, multiple check answers. Branching logic is used to reduce the number/complexity of responses. Questions were made based on literature review and consensus among the research team. The usability and functionality of the questionnaire were pilot tested with faculty members of the Division of Pediatric Gastroenterology and Hepatology at Children’s Hospital of Eastern Ontario and then revised as per suggestions.

The questionnaire was administered using Redcap Software and no passcode was needed to access the survey once they received the link. Redcap provided an automatic method for capturing and exporting responses.

### Statistics

Descriptive statistics were employed for data presentation; percentages, medians, mean, and ranges for continuous variables, and categorical variables are reported as count and numeric proportions using Stata v11.

## RESULTS

Twelve of 21 pediatric hepatologists (57%) from 12 of 13 (92%) academic centers responded, all respondents answered the full survey and none of the responses was removed from the final analysis (See Supplemental Digital Content Figure 1, http://links.lww.com/PG9/A84). In the study, 8 of 12 (66%) hepatologists were within 11 years of completing their pediatric gastroenterology and hepatology training; 3 of 12 (25%) were within 5 years of completing training. The most representation came from Ontario (n = 4, 33%), followed by Quebec (2, 17%) and Alberta (2, 17%), and 1 each from Manitoba, British Columbia, Saskatchewan, and Newfoundland and Labrador. It is comparable with the number of academic pediatric institutions in Canada, with most centers in Ontario.

### Current Clinical Care Patterns

The majority of hepatologists (11/12, 92%) see one new referral every year for FALD, with a median of 3 (range 2–10). Only 2 physicians (17%) have a multidisciplinary team to assist with post-Fontan care in their institutions, both from another province. The members of this multidisciplinary team included pediatric cardiologists, hepatologists, pulmonologists, and nurse coordinators. One hepatologist follows a patient who had a combined heart and liver transplant

### Clinic Assessment

**FIGURE 1.**
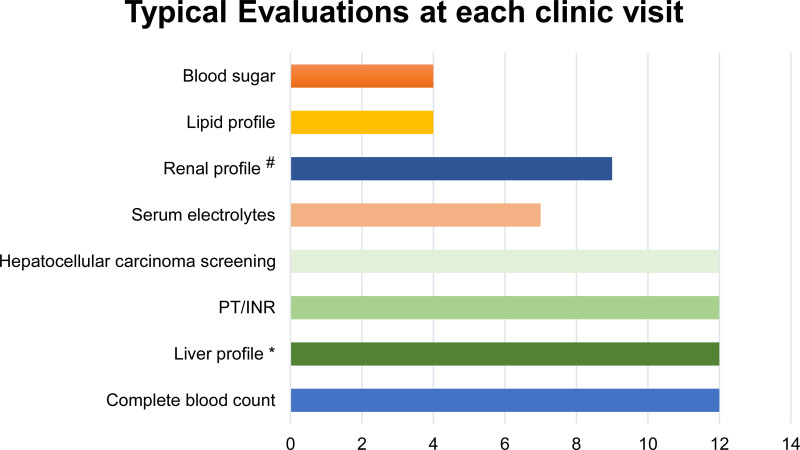
Typical blood work done during clinic visit for patients with Fontan-Associated Liver Disease. *Total and direct bilirubin, aspartate aminotransferase, alanine aminotransferase, gamma glutamyl transferase, alkaline phosphatase, albumin. ^#^Blood urea, creatinine. INR = international normalized ratio; PT = prothrombin time.

On the day of the clinic visit, the most common tests performed were complete blood count, prothrombin time/international normalized ratio, liver panel (bilirubin, aspartate aminotransferase, alanine aminotransferase levels, gamma glutamyl transferase, alkaline phosphatase, and albumin levels), and HCC screening (Fig. 1). HCC is screened for most commonly with ultrasound (12/12) and by alpha-fetoprotein assessment (AFP; 11/12), 1 respondent also uses computerized tomography (CT) or MRI abdomen to screen for HCC. At the time of the first clinic visit, 7 hepatologists (58%) conduct a serological screen for hepatitis A and B, and 6 of them also check for hepatitis C. Most respondents recommend immunization against hepatitis A and B if not already protected, and all but one would recommend repeating the hepatitis B vaccine series if serology titers do not detect immunity. Abdominal ultrasound is the most common (12/12, 100%) imaging modality used to monitor FALD progression, followed by MRI (4/12), and CT abdomen (2/12) in our study.

### Use of Liver Biopsy

Hepatologists working in 8 of 12 centers perform liver biopsy procedures in patients with FALD. In 4 centers, a biopsy is considered at the time of heart transplant evaluation and in 2 centers, it is done 10 years post-Fontan procedure or when the diagnosis of liver disease was unclear. Percutaneous liver biopsy was the most common route of doing a liver biopsy (6/8), with only 2 centers utilizing the trans-jugular route. Only 1 center utilizes congestive hepatic fibrosis score^24^ in assessing liver biopsy for FALD.

### Use of Noninvasive Markers to Assess Fibrosis

Annual transient elastography using Fibroscan is commonly used to noninvasively assess the degree of liver fibrosis (8/12, 67%). Only 1 hepatologist uses MR elastography (MRE) for fibrosis estimation at the initial consult and again at time of transition to adult care. None of the respondents uses any serum biomarkers (platelet counts, aspartate aminotransferase to platelet ratio index, Fibrosis 4 score, model for end-stage liver disease [MELD], MELD-XI, Fibro sure, Spleen size) to monitor fibrosis in their practice.

### Screening Pattern for Esophageal Varices

The timing and need of esophageal varices screening varied among the respondents, with 9 respondents agreeing that they will consider it depending on clinical status, 5 on imaging findings (nodular liver, splenomegaly, collaterals, other Doppler evidence of significant portal hypertension), 3 would consider while getting evaluated for heart transplantation and 3 centers do not perform variceal screening. Five of 12 respondents (42%) indicated that they would consider prophylactic band ligation for patients with FALD.

### Follow-Up

Five hepatologists (42%) follow FALD patients annually, 3 follow every 6 months, one follows every 3 months, one every 2–3 years, and one does not follow beyond the first visit.

### Management, Counseling, and Transition of Care

**FIGURE 2.**
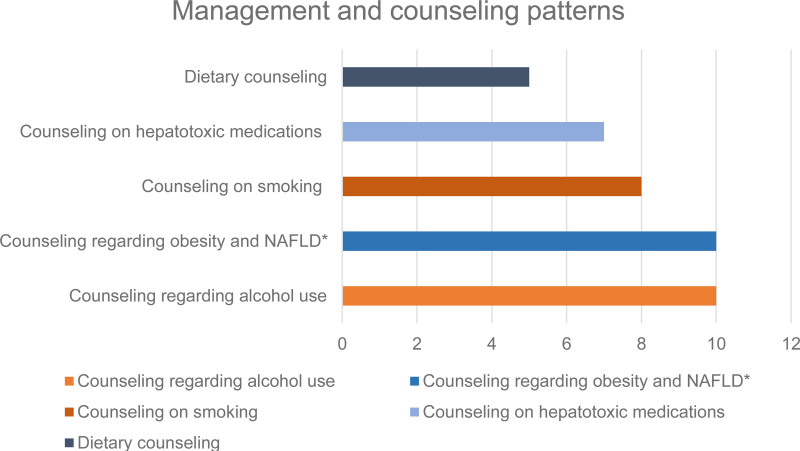
Management and counseling patterns for patients with Fontan-Associated Liver Disease. *Nonalcoholic fatty liver disease.

Ten hepatologists (83%) counsel regarding risky behavior like drugs and binge drinking, and 10 (83%) counsel on diet, obesity, and nonalcoholic fatty liver disease (Fig. 2). Seven hepatologists (58%) have a formal transition program to transfer the adult care.

## DISCUSSION

FALD comprises a wide range of structural and functional changes in the liver, including hepatic injuries, hepatic fibrosis/cirrhosis, and HCC.^6^ Our study demonstrates significant variability in the assessment and management of children with FALD among pediatric hepatologists in Canada. Variability in clinical approach and management can lead to a lack of standardization and poor outcomes.^25^ Standardizing care is essential to improve the effectiveness and quality of health care services, and to avoid preventable adverse events. Future research is necessary to develop a standardized approach to managing FALD in children.

After Fontan procedure, venous returns occur passively via the caval veins, which are connected to the pulmonary arteries. This continuous flow with elevated CVP results in chronic hepatic congestion, with the development of fibrosis over time. The low cardiac output and lymphatic overflow obstruction resulting from increased CVP hampering drainage from the thoracic duct further inflicts hepatic hypoxic injury. Natural history FALD is heterogeneous, and progression is often dependent on cardiac hemodynamics including CVP.^26^ Hepatic measurements such as venous pressure gradient have little correlation with FALD.^27^ The condition is often asymptomatic, with only a small percentage showing nonspecific symptoms underscoring the importance of ongoing hepatic monitoring in conjunction with cardiac surveillance in these patients.

The best way to address a complex, multisystemic problem that arises after Fontan is to use a multidisciplinary care strategy. According to our findings, only 2 hepatologists operate in centers with such a clinic to follow patients who have had a Fontan operation. Multidisciplinary clinics with specific institutional care pathways can lead to early diagnosis and intervention^28^; such strategies could be considered at other institutions to improve care for these patients.

Most respondents do surveillance for HCC using AFP and abdominal ultrasound in patients with FALD. Hyperplastic nodules, focal nodular hyperplasia, and HCC have been reported in patients post-Fontan, including children.^17,18,29–31^ Cirrhosis guidelines from the American Association for the Study of Liver Diseases recommend abdominal ultrasound and AFP determination every 6 months, and these guidelines should be followed until more FALD-specific data are available.^32^

In our study, annual abdominal ultrasound was the most commonly used imaging technique to monitor patients with FALD followed by MRI and CT scan. This is supported by multiple reports on FALD patients commonly presenting with abnormal imaging such as heterogeneous hepatic echotexture or liver surface nodularity, with abdominal ultrasound being the most frequently used imaging modality.^33,34^ In patients at high risk for developing HCC, MRI is the preferred method for detecting hepatic nodules due to its superior detection rate.^35^

Most respondents reported that liver biopsy was used at various time points, including at the time of assessment for heart transplantation to determine the degree of liver disease or after 10 years post-Fontan as per their institutional protocol. Percutaneous liver biopsy was the most common method of biopsy used in our study, and it was shown to be safe in previous studies in relation to FALD.^36^ It should be noted that many patients undergoing Fontan procedures may be taking anticoagulants, which can affect the safety and route of a liver biopsy. Liver biopsy is considered to be the gold standard for evaluating liver fibrosis in hepatology, including FALD. No clear consensus exists on the role or timing of liver biopsy in FALD. The authors of a recent white paper have called for liver biopsy after 10 years after Fontan and further follow-up and treatment based on the extent of fibrosis.^37^ As a means of standardizing the results of pathology, Sirius red staining, which captures portal and sinusoidal fibrosis, or the Congestive Hepatic Fibrosis Score, which captures fibrosis severity on trichome staining, are applied in FALD.^24,38^ While this scoring system is only used in one center in Canada, it could be applied more widely.

A noninvasive measure of fibrosis assessment is essential as liver biopsies are invasive, costly, and prone to error.^39^ Two-thirds of study participants assessed fibrosis using transient elastography based on Fibroscan. It is a promising modality to evaluate the evolution of liver disease in FALD, even though a precise cutoff still needs to be determined. Elastography fails to distinguish between fibrosis and congestion, leading to an overestimation of fibrosis; however, liver stiffness greater than 15 kPa likely indicates the presence of fibrosis.^40,41^ MRE is another tool to assess fibrosis and is used by 1 hepatologist in the study. MRE has shown a positive correlation with the aspartate aminotransferase to platelet ratio index, MELD, pressures in the Fontan conduit, and even histological damage.^42,43^ No serum biomarkers are used by any study participants to assess the progression of liver disease and/or fibrosis. Various serum biomarkers have been tested in Fontan patients, but have not been correlated well against histology. MELD-XI scores, however, showed some correlation with liver fibrosis severity, but no cutoff point could be determined.^44^ Liver enzyme estimation was commonly done by hepatologists in our study. Transaminase levels are typically within the normal range or mildly elevated in FALD, with gamma glutamyl transferase being the most frequently elevated enzyme.^45^

Limited adult data are reported on the prevalence of esophageal varices in patients following FALD using endoscopy,^22^ with no pediatric data. No clear consensus exists on the role of endoscopy in children with compensated liver disease. Three-fourth of respondents would consider endoscopy for screening for varices, though the necessity and appropriate intervention if varices are noted remain to be clarified. However, there have been cases of gastroesophageal variceal bleeding that report fatal outcomes; therefore, screening and timely prevention should be a priority in patients with advanced fibrosis. Children with advanced liver disease including those with varices may also need to undergo a cardiac assessment since high CVP and low arterial oxygen saturation strongly correlate with noncardiac events.^46^

In our study, almost all respondents follow patients with FALD in their practice. We found significant variability in the follow-up of patients with FALD after the initial consult, varying from every 3 months to no follow-up after initial visit, with most following annual irrespective of age/time since Fontan. The optimal timing and modality to screen for FALD have not been defined, which is reflected in our results. Time since Fontan surgery has been determined as the main risk factor for the development of FALD and is not considered by any of our study participants. The odds of developing hepatic complications increases to 4-fold in patients 11–15 years after Fontan and 9-fold after 16–20 years compared with patients <5 years postsurgery.^47^ The American Heart Association suggested hepatic surveillance every 3–4 years in children <12 years of age and every 1–2 years in ≥12 years of age.^48^ A Japanese group recommended that all children with Fontan procedure should undergo an initial assessment at 7–8 years of age, and every 6 months to a year thereafter.^49^ Because of the large heterogeneity in proposed surveillance, individual practitioner variability is higher, and a standardized strategy is urgently needed.

Our study indicates that most hepatologists discuss obesity, alcohol, hepatotoxic drugs, nonalcoholic fatty liver disease, and hepatitis A and B vaccination with children with FALD. A general liver prevention regimen should be recommended to all patients with Fontan circulation, as no specific treatment for FALD is available.

An increasing number of children with liver disease including FALD are surviving into adulthood, and formal transition programs are the need for the hour.^22^ Only 7 of 12 hepatologists in our study work at centers with a formal transition program. Every institution should provide well-designed transition services to maximize the quality of care for these patients, enabling them to control their illness and achieve their life goals.

This is the first attempt to assess the clinical approach and variability of care in patients with FALD among Canadian academic hepatologists. Therefore, we provide a solid foundation for developing a national standard, which is a strength of this study. Additionally, we provide some suggestions on monitoring and counseling patients with FALD according to a recent literature review (Tables 1, 2). An inherent limitation of surveys is that certain responses may be subjective and biased. The study also has a low number of participating centers, but this is reflective of the number of academic institutions in Canada. The response rate of the survey is comparable with other studies using mailed surveys.^50^

**TABLE 1.** Suggested monitoring and counseling in patients with Fontan-Associated Liver Disease

	Child (<10 y)	Adolescent (10–18 y)
Pediatric hepatology visit	Every 2–3 y (Can be more frequent depending on clinical status)	Annual #(Can be more frequent depending on clinical status)
Labs (see Table 2)	Yearly	Yearly
Ultrasound abdomen with liver Doppler	Every 2–3 y	Annual
Transient elastography (Can overestimates fibrosis and is important to trend in follow-up)	Every 2–3 y	Annual
Liver MRI (Add MR elastography if available)	None	Once 15 y post-Fontan, then as needed
Liver biopsy	None	Consider if undergoing heart transplant evaluation/combined liver heart transplant evaluation/advanced liver disease (No clear recommendation on role of routine liver biopsy in every patient)
Counseling on healthy lifestyle/alcohol avoidance	Physical activity as tolerated Education about NAFLD Avoidance of hepatotoxic medications	Physical activity as tolerated Education about NAFLD Avoidance of hepatotoxic medications Alcohol avoidance
Ensure vaccination against HAV and HBV	At initial visit	

HAV = hepatitis A virus; HBV = hepatitis B virus; NAFLD = nonalcoholic fatty liver disease.

**TABLE 2.** Suggested laboratory tests in patients with Fontan-Associated Liver Disease

Labs	• CBC • Electrolytes • Liver panel: total/direct bilirubin, AST, ALT, GGT, ALP albumin • PT/INR • AFP • HCC Surveillance (every 6 monthly if cirrhosis by biopsy/other noninvasive measures like imaging) • If BMI >95th percentile for children: lipid profile, glucose, insulin levels • Hepatitis serology once >10 y post-Fontan: hepatitis B surface antibody, hepatitis B surface antigen, hepatitis C antibody, hepatitis A antibody

AFP = alpha-fetoprotein; ALP = alkaline phosphatase; ALT = alanine aminotransferase; AST = aspartate aminotransferase; BMI = body mass index; CBC = complete blood count; GGT = gamma glutamyl transferase; HCC = hepatocellular carcinoma; INR = international normalized ratio; PT = prothrombin time.

In the pediatric academic hepatology care setting, we have identified variability in managing pediatric FALD. This report raises the need for continued strong collaboration between pediatric hepatologists and pediatric/adult cardiologist to develop a standardized protocol for managing and following children and youth with FALD to improve outcomes.

## ACKNOWLEDGMENT

The authors gratefully acknowledge the contributions of the members of the Canadian Pediatric Hepatology Research Group C Hepatology Committee for completing the survey.

## Supplementary Material


