# Supplementary material for: Care Pattern for Fontan-Associated Liver Disease by Academic Pediatric Hepatologists in Canada
Source: JPGN Rep. 2022 Jun 21;3(3):e207. doi: 10.1097/PG9.0000000000000207 (PMC10158345; doi:10.1097/PG9.0000000000000207)
Supplement: Supplementary file 1 [file pg9-3-e207-s001.pdf]

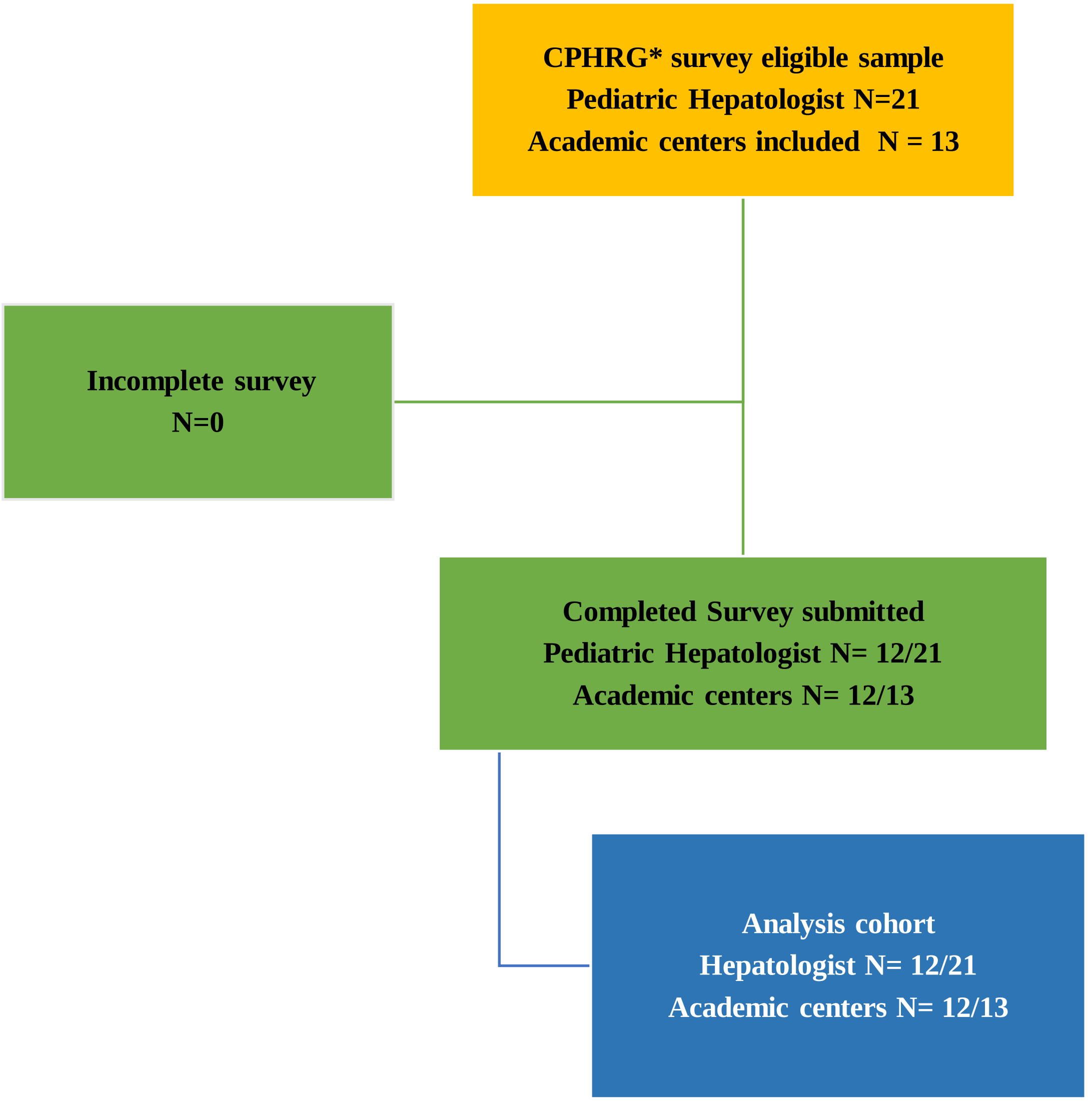

Supplementary Figure 1: Consort Diagram for the survey

*Canadian Pediatric Hepatology Research Group

**Item Category**	**Checklist Item**	**Response**
Design	Describe survey design	✓
IRB (Institutional Review Board) approval and informed consent process		✓
Development and pre-testing		✓
Recruitment process and description of the sample having access to the questionnaire		✓
Survey administration	Web/E-mail	✓
	Context	✓
	Mandatory/voluntary	✓
	Incentives	✓
	Time/Date	✓
	Randomization of items or questionnaires	X
	Adaptive questioning	✓
	Number of Items	✓
	Number of screens (pages)	X
	Completeness check	✓
	Review step	✓
Response rates	Unique site visitor	X
	Participation rate	✓
	Completion rate	✓
Preventing multiple entries from the same individual	Cookies used	Not applicable
	IP check	Not applicable
	Log file analysis	Not applicable
	Registration	Not applicable
Analysis	Handling of incomplete questionnaires	Not applicable
	Questionnaires submitted with an atypical timestamp	Not applicable
	Statistical correction	Not applicable

Supplementary Table 1: Checklist for Reporting Results of Internet E-Surveys (CHERRIES)

Dear Colleagues,

You are being invited to complete a survey for a study about “Care Pattern for Children and Youth with Fontan Associated Liver Disease in Academic Pediatric Centers in Canada: A nationwide survey of Pediatric Hepatologists”. You are being invited to join this study because as you are an academic pediatric hepatologist working in Canada. This survey is examining the care patterns of Fontan-Associated Liver Disease (FALD) in Children in Canada. In the recent years there has been growing awareness about FALD in the pediatric population in Canada and globally. As you all are aware, there are no established guidelines on screening and management of FALD in children, and there remains a need to better understand current clinical care practices for children with FALD in Canada. We hope to find out current patterns with establishment of best practice guidelines for pediatric patients with FALD. It will take about 10-15 minutes to complete.

Taking part in this study is voluntary. Your decision to participate or not in this study will not affect your standing with the CPHRG. You may choose to stop participating in the study by ending the survey. However, once you have submitted the survey, we will be unable to withdraw your information because the survey is anonymous.

All information gathered from the surveys will be kept strictly confidential, all responses will be anonymous. If we publish or present the study results, we will not use any identifying information. We will keep all survey data in secure server at CHEO. Following completion of the research study the data will be kept for 7years after the last publication of this study. They will then be destroyed.

Data collected for this research may be used in future related research projects that are either an extension of the original project or in the same general area of research (secondary use of data). Any personal identifying information will be removed from the data and cannot be linked back to you. Researchers outside of this specific study may request access to the data for new research purposes. You will not be asked to provide additional informed consent for the use of your de-identified data for future research.

You may or may not directly benefit from the study. However, your input and perspective would be of tremendous value to us.

Once the study is complete, we will share a summary report of the results with the CPHRG members, if you wish to receive a copy of the results, please contact the research team. There are no conflicts of interest to declare related to this study. By completing the survey, you are agreeing to take part in this study and agree that the data collected for this research may be used in future research within the general area of research of the current study.

The CHEO Research Ethics Board (REB) has reviewed and approved this study. Should you have any questions about your rights as a research participant, or ethical issues related to this study, you can talk to someone who is not involved in the study the CHEO REB at 613-737-7600 ext. 3272.

Please feel free to contact Dr Mohit Kehar at 613-737-7600, ext 1578 if you have any questions about the study.

Your assistance with this survey is greatly appreciated. Thank you for your time and consideration.

Dr. Mohit Kehar

Assistant Professor, Department of Pediatrics

Faculty of Medicine, University of Ottawa

Division of Pediatric Gastroenterology, Hepatology and Nutrition

Children Hospital of Eastern Ontario, Ottawa

Dr Carolina Jimenez

Associate Professor, Department of Pediatrics,

Faculty of Medicine, University of Ottawa

Division of Pediatric Gastroenterology, Hepatology and Nutrition

Children Hospital of Eastern Ontario, Ottawa

Respondent information

Date

____
(YYYY-MM-DD)

What province/territory do you practice in ?

- ◯ Alberta
- ◯ British Columbia
- ◯ Manitoba
- ◯ New Brunswick
- ◯ Newfoundland and Labrador
- ◯ Nova Scotia
- ◯ Ontario
- ◯ Prince Edward Island
- ◯ Quebec
- ◯ Saskatchewan
- ◯ Northwest territories
- ◯ Nunavut
- ◯ Yukon

Which of the following options best describes your clinical practice

- ◯ Full-time, community setting
- ◯ Part-time, community setting
- ◯ Full-time, academic setting
- ◯ Part-time, academic setting
- ◯ Other

Other

____

How long ago did you complete your training in pediatric gastroenterology and hepatology?

- ◯ < 5 years ago
- ◯ 5-10 years ago nutrition
- ◯ 11-20 years ago
- ◯ >20 years ago

Referral status and current care patterns for Fontan Associated Liver Disease

Please complete the survey below.

Thank you!

How many referral for Fontan Associated Liver Disease do you see in a year?

(Place a mark on the scale above)

Does your institution have dedicated multidisciplinary clinic for patients with post Fontan procedure including pediatric hepatology ?

- ◯ Yes
- ◯ No

Which multidisciplinary team members are involved in the care of patients post Fontan procedure ?

- ☐ Pediatric Cardiologist
- ☐ Pediatric Hepatologist
- ☐ Pediatric Pulmonologist
- ☐ Nurse practitioner
- ☐ Nurse coordinator
- ☐ Psychologist
- ☐ Registered Dietician
- ☐ Social worker
- ☐ Child life specialist
- ☐ Other

Other

____

Monitoring pattern

Please complete the survey below.

Thank you!

How frequently do you follow patients with FALD?	◯ Depends on age of the child /time from the Fontan Procedure<br>◯ Monthly<br>◯ Every 3 month<br>◯ Every 6 month<br>◯ Annual<br>◯ Every 2-3 year<br>◯ I don't follow after initial consultation<br>(FALD: Fontan Associated Liver Disease)
Please mention follow up frequency for patient < 5yr of age.	◯ Monthly<br>◯ Every 3 month<br>◯ Every 6 month<br>◯ Annual<br>◯ Every 2-3 year<br>◯ Other
Other	__________________________________
Please mention follow up frequency for patient aged between 5-10yrs.	◯ Monthly<br>◯ Every 3 month<br>◯ Every 6 month<br>◯ Annual<br>◯ Every 2-3 year<br>◯ Other
Other	__________________________________
Please mention follow up frequency for patient aged between 10-18yrs.	◯ Monthly<br>◯ Every 3 month<br>◯ Every 6 month<br>◯ Annual<br>◯ Every 2-3 year<br>◯ Other
Other	__________________________________

Assessment patterns

Please complete the survey below.

Thank you!

What is the typical Blood work you at each clinic visit for patient with FALD?	☐ CBC<br>☐ Electrolytes<br>☐ Renal profile : Creatinine level, BUN<br>☐ Liver panel: Total/direct bili, AST, ALT, GGT, Alk Phos, Albumin<br>☐ PT/INR<br>☐ Lipid panel<br>☐ Blood Glucose<br>☐ Other<br>(FALD: Fontan Associated Liver Disease)
Other	__________________________
Do you routinely screen your patient's for following viral infections at clinic visit?	☐ HCV<br>☐ HAV<br>☐ HBV<br>☐ Other
Other	__________________________
Please mention at which clinic visit you screen for Hepatitis A immune Status?	○ First clinic visit irrespective of age<br>○ 10yr of age<br>○ 15yr of age<br>○ Others
Other	__________________________
Please mention at which clinic visit you screen for Hepatitis B immune Status	○ First clinic visit irrespective of age<br>○ 10yr of age<br>○ 15yr of age<br>○ Others
Others	__________________________
Do you recommend HAV and HBV vaccinations if not immunized/protected ?	○ Yes<br>○ No
Do you recommend repeating the HBV vaccine series if found to have non-protective serology ?	○ Yes<br>○ No<br>(Non protective serology : anti-HBs below 10 mIU/mL)
Do you screen for Hepatocellular carcinoma (HCC) in patients with FALD?	○ Yes<br>○ No<br>(FALD: Fontan Associated Liver Disease)

How often do you screen for HCC ?	◯ Once<br>◯ Every 3 month<br>◯ Every 6 month<br>◯ Annual<br>◯ Other
Other	__________________________
How do you screen for HCC (mark all)	☐ Alfa-feto protein<br>☐ Ultrasound<br>☐ CT abdomen<br>☐ MRI abdomen<br>☐ Other
Others	__________________________
When you see patients with FALD in clinic, do you screen for the following?	☐ Risky Behaviour like Illict drugs, alcohol, smoking, binge drinking. (Depending on age)<br>☐ Obesity and NAFLD<br>(FALD: Fontan Associated Liver Disease)

Imaging pattern and Non Invasive monitoring in patient with FALD

Please complete the survey below.

Thank you!

Imaging Patterns

How often do you use ultrasound to monitor FALD?

◯ Once
◯ Every 3 month
◯ Every 6 month
◯ Annual
◯ Other
◯ Never
(FALD: Fontan Associated Liver Disease)

Other

____

Do you perform other imaging technique in patients of FALD apart from Ultrasound?

◯ Yes
◯ No
(FALD: Fontan Associated Liver Disease)

Which imaging technique do you use apart from Ultrasound?

☐ CT abdomen
☐ MRI abdomen
☐ MRI liver
☐ Other

Other

____

How often do you use this technique to monitor FALD ?

◯ Once
◯ Every 3 month
◯ Every 6 month
◯ Annual
◯ Other
(FALD: Fontan Associated Liver Disease)

Other

____

Non Invasive Monitoring of Fibrosis

Do you use Fibroscan (R)- transient elastography to assess for liver fibrosis in patients with FALD	◯ Yes<br>◯ No<br>(FALD: Fontan Associated Liver Disease)
How frequently do you monitor transient elastography values?	◯ Once<br>◯ Every 3 month<br>◯ Every 6 month<br>◯ Annual<br>◯ Every 2-3 year<br>◯ Other
Other	__________________________________
Do you use Ultrasound Elastography to assess for liver fibrosis in patients with FALD?	◯ Yes<br>◯ No<br>(FALD: Fontan Associated Liver Disease)
How frequently do you monitor Ultrasound Elastography values?	◯ Once<br>◯ Every 3 month<br>◯ Every 6 month<br>◯ Annual<br>◯ Every 2-3 year<br>◯ Other
Other	__________________________________
Do you use MR Elastography to assess for liver fibrosis in patients with FALD?	◯ Yes<br>◯ No<br>(FALD: Fontan Associated Liver Disease)
How frequently do you monitor MR Elastography values?	◯ Once<br>◯ Every 3 month<br>◯ Every 6 month<br>◯ Annual<br>◯ Every 2-3 year<br>◯ Other
Other	__________________________________
Do you use any other noninvasive/ serum markers for noninvasive monitoring fibrosis in patients with FALD?	◯ Yes<br>◯ No<br>(FALD: Fontan Associated Liver Disease)

Which noninvasive/ serum markers do you use for noninvasive monitoring fibrosis in patients with FALD? (check all)	☐ Platelets counts<br>☐ Liver enzymes<br>☐ APRI<br>☐ FIB-4<br>☐ MELD<br>☐ Child score<br>☐ MELD-XI<br>☐ Fibrosure<br>☐ Spleen size<br>☐ Other<br>(FALD: Fontan Associated Liver Disease)
Other	__________________________

Liver Biopsy and Esophageal varices screen

Please complete the survey below.

Thank you!

Liver Biopsy

Do you do liver biopsies in patients with FALD at your centre?	◯ Yes<br>◯ No<br>(FALD: Fontan Associated Liver Disease)
When do you consider obtaining liver biopsy in patient with FALD as per protocol at your centre?	☐ 10 yrs after Fontan procedure<br>☐ 15yrs after Fontan Procedure<br>☐ When considering heart transplant evaluation<br>☐ Other<br>(FALD: Fontan Associated Liver Disease)
Other	______________________________
How is liver biopsy performed in you center in patients with FALD?	☐ Percutaneous<br>☐ Trans jugular<br>☐ Along with Cardiac catheterization via transjugular route<br>(FALD: Fontan Associated Liver Disease)
Does your centre use special stain/scoring system to assess liver biopsy in patients with FALD?	◯ Yes<br>◯ No<br>(FALD: Fontan Associated Liver Disease)
Which special stain/Scoring system is used in your centre	☐ Sirus red stain<br>☐ Congestive hepatic fibrosis score<br>☐ Other
Other	______________________________

Esophageal Varices Screening Pattern

Do you have protocol at your centre for variceal screening in patients with FALD?

- ◯ Yes
- ◯ No

(FALD: Fontan Associated Liver Disease)

When do you consider obtaining Upper GI endoscopy for variceal screening in patient with FALD at your center?

- ☐ 10 yrs after Fontan procedure
- ☐ 15yrs after Fontan Procedure
- ☐ When considering heart transplant evaluation
- ☐ Depending on imaging result (cirrhosis/PHT)
- ☐ Depending on biopsy results
- ☐ Depending on clinical findings
- ☐ Other
- ☐ Do not perform endoscopy for variceal screening in patients with FALD

(FALD: Fontan Associated Liver Disease)

Other

____

Do you perform Prophylactic Endoscopic variceal ligation (EVL) /Sclerotherapy in patients with FALD?

- ◯ Yes
- ◯ No

(FALD: Fontan Associated Liver Disease)

Treatment patterns

Please complete the survey below.

Thank you!

63)	Do you prescribe dietary changes at time of counselling?	◯ Yes<br>◯ No
64)	Do you counsel about avoidance of alcohol?	◯ Yes<br>◯ No
65)	Do you counsel about prevention of obesity and NAFLD?	◯ Yes<br>◯ No
66)	Do you counsel about avoidance of smoking?	◯ Yes<br>◯ No
67)	Do you counsel about high dose acetaminophen and other hepatotoxic medications?	Yes<br>◯ No<br>◯
68)	Do you follow patients who had undergone Combined heart and liver transplantation (post Fontan) for cardiac and hepatic decompensation?	◯ Yes<br>◯ No
69)	Do you follow patients who had undergone isolated liver transplantation for FALD?	◯ Yes<br>◯ No<br>(FALD: Fontan Associated Liver Disease)

Transition of care

Please complete the survey below.

Thank you!

---

70) Do you have a formal program to transition your FALD patients to adult hepatologist ?

◯ Yes
◯ No
(FALD: Fontan Associated Liver Disease)
